# Metformin reduces prostate cancer risk among men with benign prostatic hyperplasia: A nationwide population‐based cohort study

**DOI:** 10.1002/cam4.2025

**Published:** 2019-04-09

**Authors:** Yu‐Jui Kuo, Fung‐Chang Sung, Po‐Fan Hsieh, Hui‐Ping Chang, Kun‐Ling Wu, Hsi‐Chin Wu

**Affiliations:** ^1^ Department of Traditional Chinese Medicine Tainan Municipal Hospital (Managed by Show Chwan Medical Care Corporation) Tainan Taiwan; ^2^ Department of Applied Cosmetology National Tainan Junior College of Nursing Tainan Taiwan; ^3^ Department of Health Services Administration China Medical University Taichung Taiwan; ^4^ Department of Urology China Medical University Hospital, China Medical University Taichung Taiwan; ^5^ Department of Family Medicine Tainan Municipal Hospital (Managed by Show Chwan Medical Care Corporation) Tainan Taiwan

**Keywords:** benign prostate hyperplasia, diabetes, metformin, prostate cancer, traditional Chinese medicine

## Abstract

Benign Prostate Hyperplasia (BPH) has been associated with prostate cancer prevalent among men after 50 years of age, however, it is unclear whether the antidiabetic drug, metformin, can reduce prostate cancer for men with BPH. The insurance claims data of men aged 50 years or older, with both type 2 diabetes mellitus (T2DM) and BPH diagnosed from 1997 to 2007 were analyzed. Individuals were followed up for at least 5 years. We identified 2906 and 2906 patients as the metformin cohort and nonmetformin cohort, respectively. The Cox method analysis showed that the metformin cohort had an adjusted hazard ratio (aHR) of 0.69 (95% confidence interval [CI] = 0.49‐0.96, *P* = 0.0298) for prostate cancer, compared to the nonmetformin cohort after controlling for age, traditional Chinese medicine (TCM) use, prostate specific antigen, and Charlson comorbidity index. Patients using TCM for BPH (per 6 months) also had an aHR of 0.41 (95% CI = 0.24‐0.69; *P* = 0.0009). In conclusion, both metformin medication and TCM use could be associated with reduced risk of prostate cancer for men with BPH and diabetes.

## 1 INTRODUCTION

Prostate cancer is the most common cancer and one of the leading causes of death in men, with an estimated 1.6 million cases and 366 000 deaths worldwide in 2015.^1^ The prevalence of prostate cancer increased with age and rose dramatically after 50 years of age.^2^ It has been predicted that the number of prostate cancer in men over 65 years would have a fourfold increase globally from 2000 to 2050.^3^

Benign Prostate Hyperplasia (BPH) is also a very common disease among elderly men affecting 50%, 70% and 90% of the men in their 50s, 60s, and 80s and above, respectively.^4^, ^5^ It costs approximately US $3.9 billion in the United States and £180 million in the UK for the disease burden caused by BPH.^6^ Epidemiological and genetic evidences have shown a strong relationship between BPH and prostate cancer. Approximately 83% of the prostate cancer patients appear with the presence of BPH indicating a greater risk of developing prostate cancer for BPH patients.^7^, ^8^ Recently, a longitudinal study conducted in Taiwan also supported the association between BPH and subsequent prostate cancer.^9^

Metformin, an oral antidiabetic drug, is a first line medication for the treatment of type 2 diabetes mellitus (T2DM).^10^, ^11^ Previous studies indicated that metformin might decrease the incidence of various cancers in diabetic patients, such as hepatocellular carcinoma, endometrial, pancreatic, colorectal, prostate, and breast cancer.^10^, ^11^, ^12^, ^13^, ^14^, ^15^, ^16^ Recently, a laboratory research demonstrated that metformin might inhibit the growth of benign prostatic epithelial cells.^5^ However, the effect of metformin use on BPH patients has not been well examined. It is unclear whether BPH patients with metformin medication are at reduced risk of prostate cancer.

The present study used a population data obtained from Taiwan's National Health Insurance (NHI) to perform a retrospective cohort study to evaluate the efficacy of metformin for diabetic patients who had been diagnosed with BPH. BPH patients with and without metformin therapy were compared for the occurrence of prostate cancer during the follow‐up period.

## 2 METHODS

### 2.1 Database

Metformin and nonmetformin cohorts were identified from longitudinal health insurance databases LHID2000, LHID2005, and LHID2010, which included all original claim data and registration files of 1 000 000 individuals randomly sampled from the years 2000, 2005, and 2010 Registry for Beneficiaries (N = 23.72 million) of the Taiwan NHI program, respectively. Because LHID2000, LHID2005, and LHID2010 have duplicate cases, a total of 2 886 862 beneficiaries were investigated in this study. No significant differences were observed in gender and age distributions between the sampled enrollees in these LHIDs and the whole insured population under the NHI program. The LHIDs enable researchers to obtain all medical services rendered to individuals registered to the database from the start of the NHI in 1995.^17^ The diagnoses were coded with the International Classification of Disease, Ninth Revision, Clinical Modification (ICD‐9‐CM) for the insurance claims. This study was exempted from review by the Tainan Municipal Hospital Institutional Review Board because it utilized deidentified secondary data released to the public for research purposes.

### 2.2 Study cohorts

We selected male patients in the metformin cohort and nonmetformin cohort by matching their age, propensity score for comorbidities, and index date at a ratio of 1:1. The inclusion criteria were males who were diagnosed with both of T2DM and BPH (ICD‐9‐CM codes, T2DM = 250.X and BPH = 600.X), aged 50 years or older, and with the diagnosis of BPH for the first time in their visits to ambulatory care centers (including outpatient departments of hospitals or clinics) between 1997 and 2007. The index date was assigned as the date of one year after the first day of metformin used. The index date of nonmetformin cohort was assigned by the matched cases. Patients diagnosed with any cancer or died prior to the index date were excluded in the study. Those with follow‐up period less than 1 year were also excluded.

For the validation of T2DM and BPH diagnoses, at least one of the following enrollment criteria must be met in the study: (a) one or more inpatient admissions with diagnosis or (b) three or more outpatient visits within a 6‐month period, each with a diagnosis. Regular metformin use was defined as continuous prescription for a minimum of 3 months within 1 year, and the Traditional Chinese Medicine (TCM) also used the same definition. However, Chinese medicine treated for T2DM and BPH are different and often change prescriptions and ingredients. Therefore, we can only analyze the overall effectiveness of TCM use for either T2DM or BPH. The study deleted the patients used TCM before the index date. In addition, the treatment effectiveness of Chinese medicine was also assessed. In both cohorts, comorbidities observed before the index date were identified and analyzed using the Charlson comorbidity index (CCI). Comorbidities associated with T2DM were excluded, including type 1 diabetes mellitus and diabetes associated end organ damage. The endpoint of follow‐up was the date of prostate cancer diagnosis, death, terminated enrollment from the NHI, or the end of 2012. Follow‐up data were available for a minimum of 5 years for all subjects.

### 2.3 Identification of prostate cancer

We retrieved information from the Catastrophic Patient Database for the diagnosis of prostate cancer (ICD‐9‐CM = 185) and all other cancers because all patients registered in this database have been carefully reviewed providing accurate diagnoses. Owing to the high cost of cancer treatment, strict guidelines are applied to carefully examine medical records before a patient meets the entry requirements. Successfully enrolled patients must have sufficient evidences supporting their cancer diagnosis, such as histological or pathological reports, laboratory evidences and clinical images. The clinical images may include tumor markers, X‐rays, bone scans, computed tomography or magnetic resonance imaging. Furthermore, at least two oncologists must comprehensively examine each patient's medical records and laboratory information, including images.

### 2.4 TCM

Traditional Chinese Medicine (TCM) for the treatment of BPH includes Huang Qin,^18^ Fu Ling,^19^ Ze Xie,^20^ Huang Bo,^21^ and Zhi Mu.^22^ TCM for the treatment of T2DM includes San Qi,^23^ Shu Di Huang,^24^ Shan Yao,^25^ Shan Zhu Yu,^26^ Mu Dan Pi.^27^ In this study, we estimated the dosage of unilateral TCM to provide a reference for the calculation of prescription doses. Appendix Table S1 provides a list of all the TCMs considered in this study.

### 2.5 Statistical analysis

The SAS system (SAS System for Windows, Version 9.4, SAS Institute Inc, Cary, NC) was used for statistical analyses. We performed *t*‐test and Chi‐square test of independence to compare the base line characteristics between metformin and nonmetformin cohorts, including age, TCM uses, prostate specific antigen (PSA) test frequency and comorbidities. Numbers of incident prostate cancer in both cohorts were estimated. Cox proportional hazards regression analysis was used to calculate the metformin cohort to nonmetformin cohort crude and adjusted hazard ratio (cHR and aHR) with corresponding 95% confidence interval (CI). Metformin use (per 6 months), TCM use for DM (per 6 months), and TCM use for BPH (per 6 months) were treated as a time‐dependent covariates. The Kaplan–Meier method was used for the determination of the cumulative incidence of prostate cancer in both cohorts, and the differences between cohorts were tested with the log‐rank test. Sensitivity analyses were also performed using the Cox regression hazards model on subgroups classified by comorbidity*.*

## 3 RESULTS

**Figure 1:**
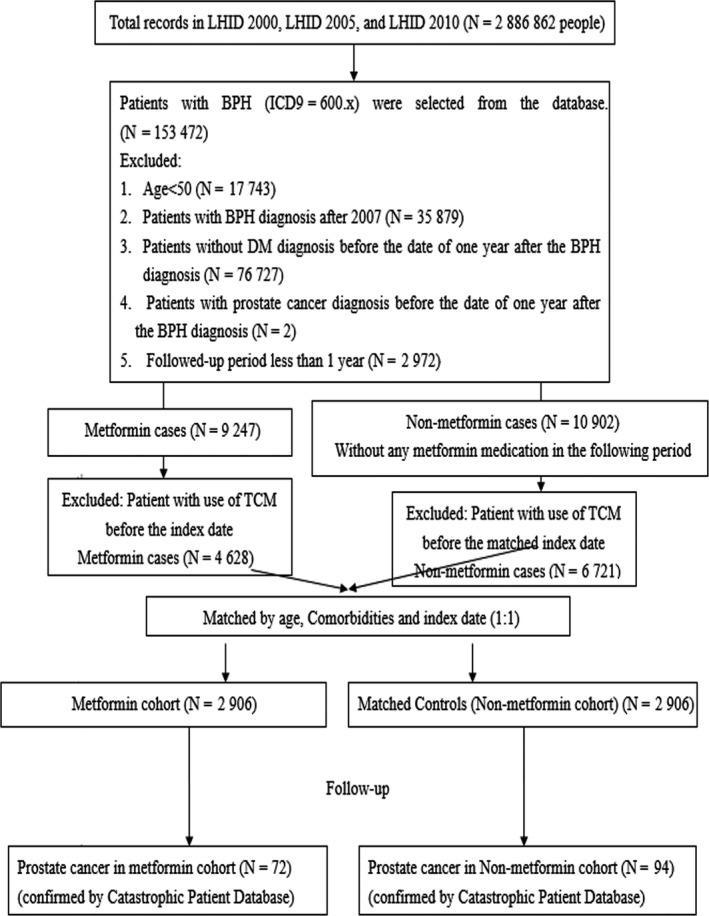
Study flow chart

**Figure 2:**
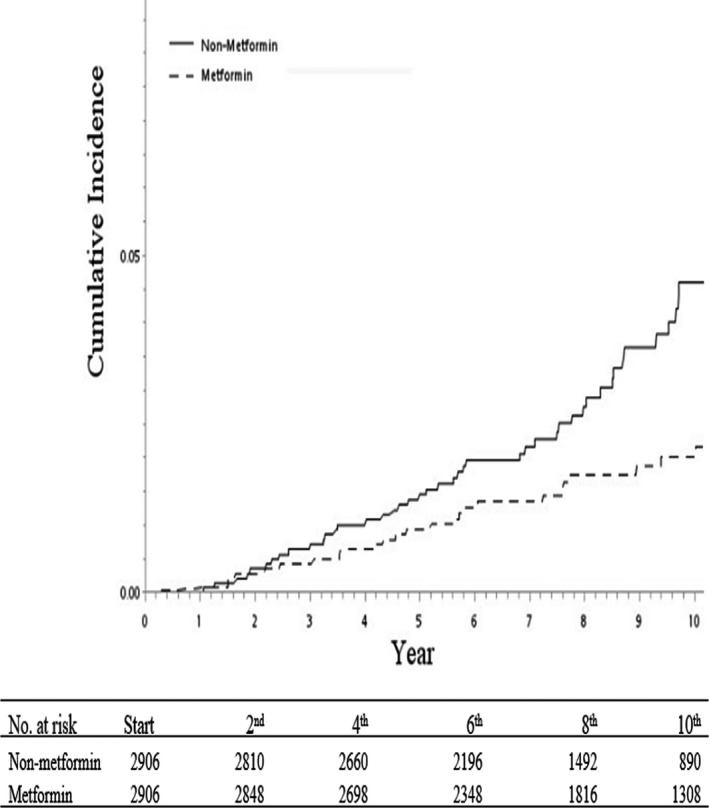
Cumulative incidence of prostate cancer among patients prescribed with metformin and nonmetformin

Between 1 January 1997 and 31 December 2007, we identified 2906 and 2906 patients in the metformin cohort and nonmetformin cohort, respectively, matching by age, CCI, retinopathy, nephropathy, neuropathy, and index date after exclusion of the ineligible subjects (Figure 1). The distributions of age, TCM for DM use, TCM for BPH use, the number of PSA screening, and CCI comorbidities were similar between both cohorts (Table 1). Most subjects were 65–80 years of age. The cumulative incidence of prostate cancer was significantly lower in the metformin cohort than in the nonmetformin cohort, as shown in the Kaplan–Meier curves (Figure 2). The study defined metformin (per 6 months) as a time‐dependent variable. In the stratified Cox model, the metformin cohort had an aHR of 0.69 (95% CI = 0.49‐0.96, *P* = 0.0039) for prostate cancer, compared to the nonmetformin cohort after the adjustment of age, TCM for BPH use, TCM for DM use, insulin usage, and CCI (Table 2). We also found that patients administrated with TCM for BPH had an adjusted aHR of 0.41 (95% CI = 0.24‐0.69; *P* = 0.0009) compared to those without.

**Table 1:** Baseline characteristics of patients with benign prostate hyperplasia compared between those with and without metformin treatment

	Nonmetformin N = 2906	Metformin N = 2906	*P*‐value
Age, y	68.03 ± 8.67	66.41 ± 8.25	<0.0001
Age group			<0.0001
50‐64	1048 (36.06)	1244 (42.81)	
65‐79	1644 (56.57)	1554 (53.48)	
≥80	214 (7.36)	108 (3.72)	
TCM for DM	1220 (50.71)	1186 (49.29)	0.3652
TCM for BPH	1256 (50.65)	1224 (49.35)	0.3961
Insulin usage	66 (2.27)	520 (17.89)	<0.0001
Severity of type 2 diabetes			<0.0001
Low	1026 (35.31)	432 (14.87)	
Midden	1026 (35.31)	2004 (68.96)	
High	854 (29.39)	470 (16.17)	
PSA (times/per year)	0.17 ± 0.32	0.18 ± 0.31	0.5999
Comorbidities			
Myocardial infarction	42 (1.45)	46 (1.58)	0.6674
Congestive heart failure	122 (4.20)	138 (4.75)	0.3100
Peripheral vascular disease	36 (1.24)	38 (1.31)	0.8150
Cerebrovascular disease	492 (16.93)	430 (14.80)	0.0260
Dementia	58 (2.00)	50 (1.72)	0.4371
Chronic lung disease	246 (8.47)	230 (7.91)	0.4441
Connective tissue disease	28 (0.96)	28 (0.96)	>0.9999
Ulcer	888 (30.56)	964 (33.17)	0.0324
Chronic liver disease	552 (19.00)	562 (19.34)	0.7389
Hemiplegia	54 (1.86)	40 (1.38)	0.1454
Moderate or severe kidney disease	158 (5.44)	176 (6.06)	0.3103
Tumor, leukemia, lymphoma	98 (3.37)	104 (3.58)	0.6674
Moderate or severe liver disease	8 (0.28)	4 (0.14)	0.2477
Malignant tumor, metastasis	0 (0.00)	18 (0.62)	—
AIDS	0 (0.00)	0 (0.00)	—
Retinopathy	54 (1.86)	52 (1.79)	0.8446
Nephropathy	42 (1.45)	40 (1.38)	0.8240
Neuropathy	88 (3.03)	72 (2.48)	0.1996
Erectile dysfunction	2750 (94.63)	2714 (93.39)	0.0466
Prostate cancer	94 (3.23)	72 (2.48)	0.0832

AIDS, acquired immune deficiency syndrome; BPH, benign prostate hyperplasia; DM, diabetes mellitus; PSA, prostate specific antigen; TCM, traditional Chinese medicine.

**Table 2:** Prediction for occurrence of prostate cancer

	Crude	*P*‐value	Adjusted	*P*‐value
	cHR		aHR	
Metformin vs Nonmetformin	0.65 (0.45‐0.93)	0.0195	0.69 (0.49‐0.96)	0.0298
Severity of type 2 diabetes				
Low	Ref.		Ref.	
Midden	0.77 (0.42‐1.42)	0.4004	0.89 (0.58‐1.37)	0.6033
High	1.09 (0.51‐2.35)	0.8217	1.27 (0.82‐1.97)	0.2938
TCM for DM	0.27 (0.14‐0.52)	0.0001	1.04 (0.62‐1.74)	0.8817
TCM for BPH	0.17 (0.08‐0.37)	<0.0001	0.41 (0.24‐0.69)	0.0009
Insulin usage	0.29 (0.09‐0.87)	0.0271	0.53 (0.25‐1.10)	0.0891
Comorbidities				
Myocardial infarction	NA		NA	
Congestive heart failure	0.33 (0.07‐1.65)	0.1785	0.51 (0.12‐2.09)	0.3514
Peripheral vascular disease	1.50 (0.61‐3.67)	0.3744	1.62 (0.40‐6.59)	0.5012
Cerebrovascular disease	NA		1.17 (0.75‐1.84)	0.4890
Dementia	1.50 (0.42‐5.32)	0.5299	NA	
Chronic lung disease	NA		0.79 (0.38‐1.62)	0.5190
Connective tissue disease	1.33 (0.46‐3.84)	0.5943	NA	
Ulcer	2.00 (0.60‐6.64)	0.2577	1.76 (1.26‐2.45)	0.0009
Chronic liver disease	0.50 (0.09‐2.73)	0.4236	1.31 (0.88‐1.95)	0.1880
Hemiplegia	NA		0.82 (0.19‐3.49)	0.7845
Moderate or severe kidney disease	1.00 (0.14‐7.10)	>0.9999	NA	
Tumor, leukemia, lymphoma	NA		2.10 (1.02‐4.30)	0.0435
Moderate or severe liver disease	NA		NA	
Malignant tumor, metastasis	NA		NA	
AIDS	NA		NA	
Retinopathy	NA		0.84 (0.20‐3.43)	0.8055
Nephropathy	0.50 (0.09‐2.73)	0.4236	5.39 (2.29‐12.68)	0.0001
Neuropathy	0.65 (0.45‐0.93)	0.0195	0.54 (0.13‐2.22)	0.3968
Erectile dysfunction	0.98 (0.54‐1.76)	0.9395	0.95 (0.52‐1.72)	0.8642

AIDS, acquired immune deficiency syndrome; BPH, benign prostate hyperplasia; DM, diabetes mellitus; TCM, traditional Chinese medicine.

Table 3 shows the results of sensitivity‐analysis, which is consistent with previous analysis. When compared to people without any kind of comorbidities, those in the metformin cohort had a significant lower hazard of developing prostate cancer than those in the nonmetformin cohort. Besides, the results fluctuated among people with comorbidities because of small cancer numbers. Owing to the sample size, the duration of cumulative medication is insufficient for the calculation of prostate cancer development.

**Table 3:** Sensitivity‐analysis for occurrence of prostate cancer

	Without disease	*P*‐value	With disease	*P*‐value
	sHR*		sHR*	
Myocardial infarction	0.62 (0.45‐0.84)	0.0021*	NA	
Congestive heart failure	0.63 (0.46‐0.86)	0.0038*	NA	
Peripheral vascular disease	0.63 (0.46‐0.86)	0.0036*	NA	
Cerebrovascular disease	0.64 (0.46‐0.89)	0.0080*	0.52 (0.22‐1.21)	0.1301
Dementia	0.61 (0.45‐0.84)	0.0020*	NA	
Chronic lung disease	0.67 (0.49‐0.92)	0.0127*	NA	
Connective tissue disease	0.62 (0.45‐0.84)	0.0022*	NA	
Ulcer	0.62 (0.41‐0.92)	0.0165*	0.59 (0.36‐0.97)	0.0392*
Chronic liver disease	0.58 (0.41‐0.83)	0.0024*	0.77 (0.39‐1.52)	0.4568
Hemiplegia	0.63 (0.46‐0.85)	0.0031*	NA	
Moderate or severe kidney disease	0.62 (0.45‐0.84)	0.0022*	NA	
Tumor, leukemia, lymphoma	0.65 (0.47‐0.89)	0.0073*	0.28 (0.06‐1.43)	0.1258
Moderate or severe liver disease	0.61 (0.45‐0.84)	0.0021*	NA	
Malignant tumor, metastasis	0.62 (0.45‐0.84)	0.0023*	NA	
AIDS	NA		NA	
Retinopathy	0.62 (0.45‐0.84)	0.0023*	NA	
Nephropathy	0.66 (0.48‐0.90)	0.0091*	NA	
Neuropathy	0.59 (0.43‐0.81)	0.0011*	NA	
Erectile dysfunction	0.30 (0.09‐1.03)	0.0558	0.65 (0.47‐0.90)	0.0083*

AIDS, acquired immune deficiency syndrome; sHR, subdistribution hazard ratios.

* *p* < 0.05.

## 4 DISCUSSION

Metformin is an effective antidiabetic drug for T2DM treatment. Diabetes is associated with increased risk of BPH,^28^ and BPH could be a factor associated with the development of prostate cancer.^7^ Metformin has been associated with a reduced risk of prostate cancer,^14^, ^15^, ^29^ but the relationship with metformin in BPH is unclear. One in vitro study suggested that metformin may repress the growth of benign prostatic epithelial cells by inhibiting insulin‐like growth factor 1 (IGF‐1) and IGF‐1 receptor (IGF‐1R) secretion in stromal cells.^5^ However, population‐based study of metformin efficacy on BPH was limited because it is difficult to perform a randomized population trial. We used insurance claims data to perform a population‐based retrospective cohort study. In the present study, we found that most of the diabetic patients with BPH were elderly men aged 66–80 years. Our findings showed that metformin could reduce the risk of prostate cancer by up to 40% in diabetic patients with BPH. Moreover, TCM use is also associated with a reduced risk of developing prostate cancer.

Our study results consistently support previous studies. Epidemiologic evidence show that the prevalence of BPH increases with age, from 20% to 50%, 70% and 90% in men in their 40s, 50s, 60s, and 70s or older.^4^, ^5^, ^30^ The BPH patients in our study were mostly 65–79 years old. Furthermore, our study is the first one using BPH to investigate the association between metformin therapy and prostate cancer development. We found that metformin use was associated with a decreased risk of prostate cancer in BPH males. This finding was in accordance with several studies. A systematic review and meta‐analysis stated that prostate cancer recurrence may be lowered by the use of metformin.^14^ One retrospective cohort study reported that a longer duration of metformin use was associated with lower incidence of prostate cancer among diabetic patients diagnosed with high‐grade prostatic intraepithelial neoplasia.^31^ In Switzerland, a multicenter clinical trial was conducted on nondiabetic patients with prostate cancer. The subjects were prescribed 1000 mg of metformin twice daily. The results showed that 36% of the subjects were progression‐free at 12 weeks.^32^ Metformin may have broader clinical application as a new medication for cancer prevention due to its low cost and safety.^33^ Therefore, metformin may be beneficial for the treatment of BPH and the prevention of prostate cancer.

Some studies are inconsistent with ours. For example, population studies in the UK and Canada failed to find the benefit of metformin in reducing prostate cancer risk in patients with T2DM.^34^, ^35^ Chen et al have concluded in a systematic review, that metformin use was not associated with the risk of prostate cancer in both Western men and Asian men.^36^ Another study reported that metformin use could not reduce mortality from prostate cancer and from all causes.^37^

Typical symptoms of BPH include urinary hesitancy, weak stream, nocturia, incontinence, and recurrent urinary tract infections. Many interventions are effective in relieving these symptoms, such as alpha blockers, 5‐alpha reductase inhibitors, transurethral resection of the prostate and laser‐based surgeries.^30^ Recently, healthy diet and lifestyles have been considered as optimal strategies to prevent BPH.^38^ In the present study, our finding reflects that metformin is a useful medicine for BPH patients in reducing prostate cancer risk. Cancer prevention of metformin is effective through the mechanism by decreasing hyperinsulinemia‐associated carcinogenesis.^39^ Anwar et al suggested that metformin amends hyperglycemia by reducing gluconeogenesis and ameliorates metabolic syndromes, endothelial function, oxidative stress, insulin resistance and fat redistributionin T2DM patients.^29^ However, the exact mechanism responsible for the effect of metformin on BPH and prostate cancer needs further elucidation.

We studied the effectiveness of TCM on BPH patients with diabetes for several reasons. First, the Taiwan NHI system covered costs of Chinese medicine services since 1995, and over 60% of the people had used TCM in Taiwan.^40^, ^41^ Second, several TCM herbs are effective for treating T2DM and BPH, such as Huang Qin, San Qi, Shu Di Huang, and Shan Yao.^42^, ^43^, ^44^, ^45^ Third, more than 1500 plants that have been used in Chinese herbal medicines contain antioxidant properties.^46^, ^47^ The antioxidant activity may suppress the progress of BPH and prevent the development of prostate cancer. Fourth, TCM is a popular medication in BPH patients with diabetes. However, TCM treated for BPH and T2DM are different. Besides, different TCM physicians often use different medicine and prescriptions. Therefore, we only analyze the overall effectiveness of TCM use for either T2DM or BPH.

The present study revealed that patients prescribed with TCM for the treatment of BPH had nearly 60% reduced risk of developing prostate cancer compared to those without. The mechanism could be related to TCM's antioxidant activity. Previous study suggests that some herbal agents can relieve symptoms of BPH including saw palmetto berry, rye grass pollen extract, and pygeum.^30^, ^48^ Medications with metformin plus Chinese medicine have a better therapeutic outcome than using either metformin or TCM alone. Integrative medication with a combination of Western and Chinese medicine may be useful to prevent prostate cancer in BPH patients.

TCM contains estrogenic chemicals which have been used to treat the symptoms of cancer. The effective chemicals are produced by phytoestrogens, herbs, leaves, rhizome or roots, and extracts of fruit in TCM.^49^ Several evidence showed that an increasing prostate cancer risk is associated with decreasing estradiol levels.^50^, ^51^ Paradoxically, estrogens are effective against androgen‐dependent prostate cancer, but might also be associated with the presence of malignancy.^52^ Men with elevated plasma estrogens were more likely to have an increased risk of prostate cancer.^51^, ^53^ In the present study, some ingredients of TCMs have been proved to have micro amounts of estrogens, including Fu Ling,^19^ Huang Bo,^21^ Zhi Mu,^22^ San Qi,^23^ Shu Di Huang,^24^ Shan Yao,^25^ and Mu Dan Pi.^27^ However, we were unable to estimate the influence of estrogens generated by TCMs because the relative sample size is too small.

This study is the first to use the nationwide population data to evaluate the treatment effectiveness of metformin to prevent prostate cancer for men with BPH and diabetes. To the best of our knowledge, this study covers the largest sample size compared to other similar studies. Our observation provides the strongest data available to date. Furthermore, men with BPH and diabetes are at an elevated risk of developing prostate cancer. Because metformin can benefit men with high prostate cancer risk, the medication might be also effective for men with low risk.

This study has several limitations. First, selection bias is an inherent limitation in the nature of retrospective study. To compensate for this disadvantage, we used full and accurate data retrieved from the NHI program and selected the case and control groups by matching their age, commorbidies, and index date. Second, the NHI program did not contain personal information regarding disease severity, lifestyles, family medical history, or body mass index. To cover this shortage, we adjusted CCI of comorbidies to assure no systematic difference in baseline conditions between the two cohorts. Third, our subjects were BPH patients with T2DM, future studies for nondiabetic patients or healthy population are required. Fourth, unfortunately, many TCMs can be obtained from the OTC (over the counter) with out‐of‐pocket payment precluding academic assessment of the overall usage in these groups of East Asian patients. Finally, we only identified 2906 patients in the metformin cohort; the sample size may not be large enough to estimate the best dosage of metformin therapy and the effectiveness of estrogens generated by TCM. Future studies seek to evaluate effective dosages of metformin and estrogenic chemicals in TCM for BPH patients for prostate cancer prevention.

## 5 CONCLUSIONS

Our data show that the metformin medication may reduce prostate cancer risk for in 40% the diabetic patients with BPH. Limited men with BPH are metformin users. Those who use Chinese medicine also have benefited from the treatment. It is reasonable to assume that the early initiation of metformin treatment may further reduce the prostate cancer risk. Future studies to evaluate the most suitable dosage of metformin for BPH patients are essential.
