# Supplementary material for: Metformin reduces prostate cancer risk among men with benign prostatic hyperplasia: A nationwide population‐based cohort study
Source: Cancer Med. 2019 Apr 9;8(5):2514–23. doi: 10.1002/cam4.2025 (PMC6536940; doi:10.1002/cam4.2025)
Supplement: Supplementary file 1 [file CAM4-8-2514-s001.docx]

**Appendix Table 1: List of Traditional Chinese Medicine**

| Chinese name | Composition (Pinyinname) | Composition (Latinname) |
| --- | --- | --- |
| For BPH |  |  |
| 黃芩 | Huang Qin | Radix Scutellariae |
| 茯苓 | Fu Ling | Poria |
| 澤瀉 | Ze Xie | Rhizoma Alismatis |
| 黃柏 | Huang Bo | Cortex Phellodendri |
| 知母 | Zhi Mu | Rhizoma Anemarrhenae |
| For DM |  |  |
| 三七 | San Qi | Radix NotoGinseng |
| 熟地 | Shu Di Huang | Radix Rehmanniae Praeparata |
| 山藥 | Shan Yao | Rhizoma Dioscoreae |
| 山茱萸 | Shan Zhu Yu | Fructus Corni |
| 牡丹皮 | Mu Dan Pi | Cortex Moutan Radicis |

Note: BPH=Benign Prostate Hyperplasia; DM= Diabetes Mellitus
